# Defining the islet: a minimum size threshold and opportunities for standardization

**DOI:** 10.3389/fendo.2026.1927749

**Published:** 2026-09-30

**Authors:** Alex M. Tollefson, Manami Hara

**Affiliations:** Department of Medicine, The University of Chicago, Chicago, IL, United States

**Keywords:** 2D analysis, 3D analysis, islet, statistics, vascularization

## Abstract

Although islets play a central role in the pathogenesis of diabetes, they still lack a universally accepted definition beyond being large clusters of endocrine cells within the pancreas. Previous definitions have primarily relied on arbitrary size or cell-count thresholds rather than biologically meaningful criteria. As a result, this longstanding ambiguity has recently prompted the introduction of the term endocrine objects (EOs), which replaces the traditional distinction among islets, small endocrine cell clusters, and solitary endocrine cells with a unified, continuous size-based classification. This raises a fundamental question: what biologically meaningful features distinguish islets from their smaller endocrine counterparts? In our recent study of volumetric intraislet vascularization among various mammalian species including humans, we defined the smallest islet size that receives direct internal vascularization as being ~40 µm in diameter. Considering that the islet has been described as highly vascularized, the presence or absence of direct vascularization should fairly distinguish islets from non-islet single endocrine cells and small cell clusters. In addition to variability in islet size thresholds, the lack of standardized analytical approaches has further complicated meaningful comparisons across islet studies. This perspective aims to address these limitations through discussion of islet effective diameter, islet sampling, and comparing 2D and 3D analytical methods.

## Introduction

A newly emerged term, endocrine objects (EOs), was first defined and proposed by Murrall et al. in a preprint in April 2025. Prior to their formal publication in November 2025 (1), Drotar et al. incorporated the term EOs in their study without its definition nor rationale (2). A commentary on Drotar et al. by Homann amusingly titled “Sweating the Small Stuff” appears to support the use of EOs over “the more obvious choice of pancreatic islet,” due to a lack of current consensus about the size threshold for the islet (3). In the past, non-islet endocrine cells have been excluded by applying various size thresholds: including minimum diameter cutoffs of 25 µm (4), 30 µm (5), 35 µm (2), 50 µm (6, 7), and 70 µm (8). By contrast, other studies have included non-islet endocrine cells in analysis (9–12). Such disagreement has hindered precise comparisons across islet studies.

In our recent 3D analysis revisiting pancreatic vasculature across various mammalian species including humans, we identified an islet size threshold of ~40 µm in effective (sphere-normalized) diameter based on the presence of direct internal vascularization (13). Endocrine cells below this threshold, as observed histologically, are primarily present as single cells or small clusters and, consistent with most pancreatic cell types, maintain close association with capillary networks rather than receiving direct internal vascularization. Distinction based on vasculature is therefore biologically meaningful, readily quantifiable, and closely aligns with the longstanding recognition of dense intraislet capillary networks (14–16). In this perspective, we will first describe how we determined the smallest vascularized islets, which may help preserve the historical and respected term, the islet of Langerhans. In further discussion of standardization, we will also discuss technical aspects of islet detection in 2D and 3D, graphical presentation, and approaches to statistical analysis.

## Definition of the islets

Through our recent work, we have shown that the islets are not enclosed structures, such as having an individual independent blood supply separated from the surrounding exocrine tissues (14, 17–21). Rather, the pancreatic vasculature is entirely integrated between the endocrine (islets) and exocrine pancreas (22, 23). Intravital imaging of the flow direction of fluorescent-labeled red blood cells at the peri-islets (n=42) in MIP-GFP mice (24) demonstrated that the ratio of the inward and outward blood flow was 1:1 (22). Further, in our recent study on volumetric intraislet capillary density, we quantified the exact number of individual capillaries intersecting the islet margins by isolating capillaries that passed through the boundaries of digitally rendered islets (**Figure 1A**) (13). Smaller clusters of endocrine cells associate with nearby capillaries, but they are not directly and internally vascularized as islets are (**Figure 1B**). Counts of capillaries intersecting the islet surface had a linear correlation with the islet surface area (**Figure 1C**) which, beyond supporting the integrated structure of pancreatic vasculature, demonstrates that the degree of vascular integration increases with islet size. Plotting the islet effective diameter against capillary count revealed the smallest islet size that was internally vascularized as ~40 µm in diameter (**Figure 1D**). It is noted that an absolute cutoff value could modestly vary in the vicinity of 40 µm even at the level of individual subjects, and it is worth expanding studies to diverse species. Notably, present use of vascular criteria to more clearly differentiate islets from smaller endocrine cell clusters does not require subsequent analyses to use vascular stains to reconfirm the ~40 µm threshold. Provided that other works corroborate this vascularly related size, it establishes a reliable rationale for what constitutes an islet.

**Figure 1:**
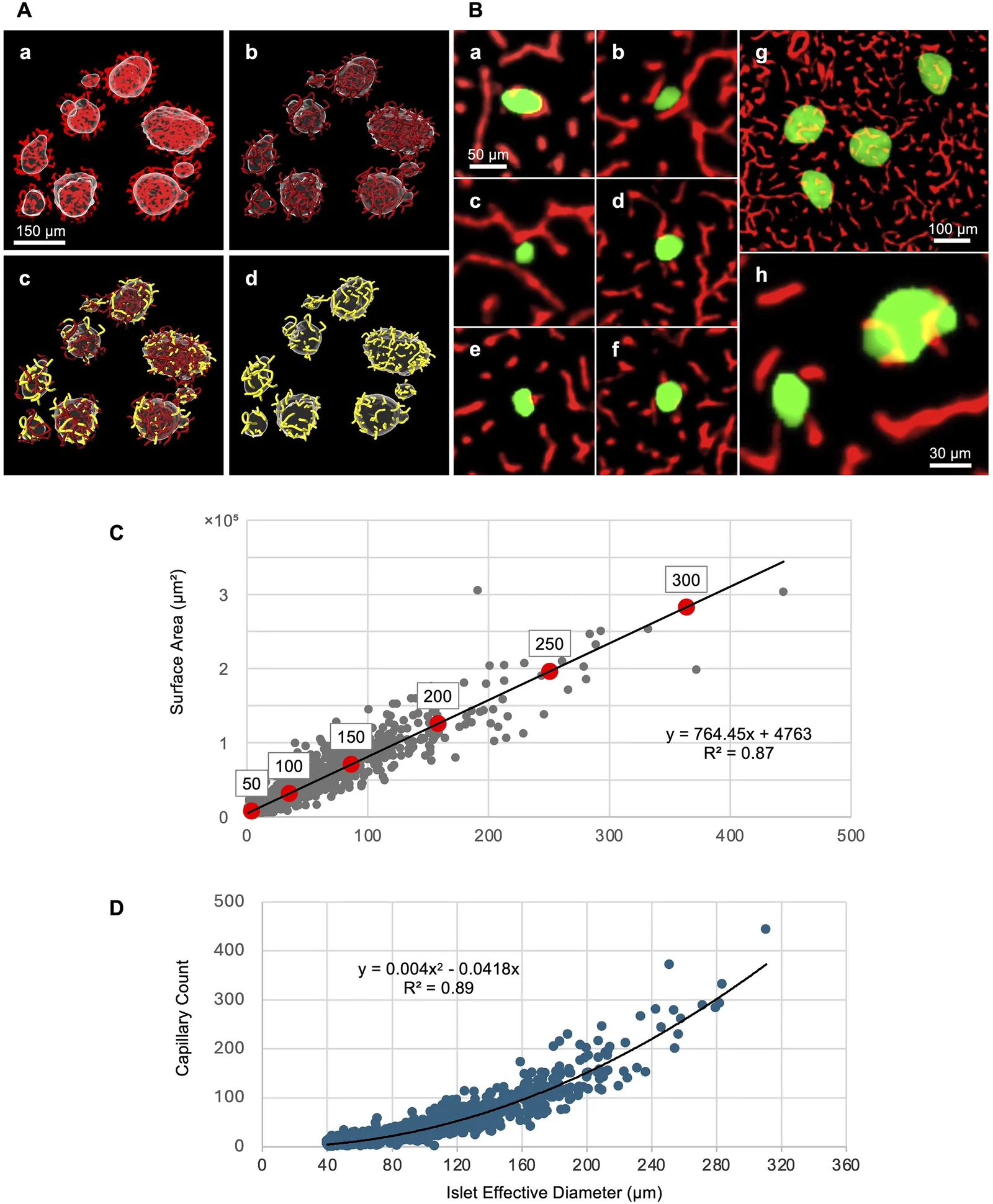
**(A)** Approach to quantifying the number of individual capillaries at the islet periphery. **a**. Capillary fluorescence inside and 25 μm outside of rendered islets: CD31 (red). Scale bar: 100 µm. **b**. Rendered capillary segments. **c**. Highlighted segments at the islet periphery, intersecting their margins. **d**. Isolation of highlighted segments for quantification. **(B)** Vascular distribution with respect to endocrine structures. **a–f**. Non-islet single endocrine cells and small clusters (<40 μm), or niSSCs, in association with capillaries: pan endocrine (green) and CD31 (red). **g**. Islets exhibiting direct, internal vascularization. **h**. Comparison of an niSSC and islet. **(C)** Count of capillary segments on the islet margins plotted against islet surface area (13). Numerically labeled red data points indicate islet effective diameters (µm). The ~40 µm islet size threshold value was calculated by converting the y-intercept from surface area to diameter. This represents the size at which endocrine cell clusters consistently receive direct vascularization, identifying them as islets. **(D)** Conversion of plot in B to demonstrate relationship between islet margin capillary count and islet effective diameter (13). The islet size threshold value can be informally approximated by looking at the x-intercept.

During fetal and early postnatal pancreatic development, the islet vasculature undergoes dynamic growth, maturation, and remodeling, coordinated with endocrine-cell differentiation, islet morphogenesis, and postnatal expansion (25–28). In contrast, the adult islets are relatively resilient to vascular changes, as a two-fold reduction in vasculature via VEGF-A inactivation preserved islet architecture, beta-cell gene expression, mass, and function (29). Therefore, we restrict the proposed definition to the mature pancreas. Consistent with this relative vascular stability, a large cohort study of T1D has shown that pancreatic capillaries and nerve fibers persisted in T1D despite beta cell loss (30). Our recent study on T2D also demonstrated preservation of islet capillary networks (13). In line with this, despite the dramatic reduction in beta cell volume in T1D, studies directly assessing surviving islets have found that overall size distributions are conserved (4, 5). In fact, significant loss of islet vasculature in T1D is unlikely, as such vascular depletion would be expected to cause remaining alpha- and delta-cell death and total islet atrophy. Furthermore, the use of effective diameter should help account for potential islet deformation resulting from beta-cell loss, thereby providing a more robust measure of islet size. Taken together, these considerations suggest that the proposed islet-size cutoff remains applicable and useful for the analysis of islets in T1D, although confirmatory three-dimensional (3D) analyses would further substantiate its validity.

The islet-size cutoff for direct vascularization classifies pancreatic endocrine cells into two groups: [1] *islets* as the inherited term of the islet of Langerhans (≥ 40 µm in diameter); and [2] *non-islet single endocrine cells and small clusters* (niSSC). Importantly, these two groups of endocrine cells are *not static*. For example, in T1D, the overall change in the islet size distribution may manifest as a leftward shift, occurring across all islet size ranges. When an islet that was previously >40 µm in diameter loses beta-cells, the entire islet mass progressively decreases, accompanied by loss of its internal vascularization, ultimately becoming classified as a niSSC. It is critical to distinguish intraislet vascularization from the mere physical association of niSSCs with adjacent capillaries.

## Contribution of extra-islet endocrine cells to the total endocrine area

Existence of single and small clusters of endocrine cells was already documented by Paul Langerhans in 1869 (31). It has subsequently been described by many investigators throughout the 1980s and 1990s (reviewed in 3). Among such studies, Kaiho et al. quantified the contribution of “beta-cell–rich extra-islet cells (<30 μm diameter)” to the total endocrine area in 1986 (32) and reported that despite their high counts (>80%), they contributed to <1% of the total endocrine area. This is in agreement with our prior findings, as we have examined the entire range from single endocrine cell to largest islet to account for size-dependent differences in endocrine content (10, 11). Recent histological analyses, however, both in 2D (1, 2) and 3D (5, 33), collectively reported that “small EOs” constitute a substantial proportion of total pancreatic endocrine content (~40–60% in terms of numbers, and 16–20% in terms of total endocrine area). It is overall quite understandable for those studies to have resulted in such outstanding numbers, when there has been no consensus about “what size of endocrine structure observed in histological tissue sections in fact constitutes an islet” (3). As a consequence of this lack of clarity, individual studies have applied different criteria to exclude and/or include the smallest cells or clusters. Further, when they categorized small EOs (against medium and large EOs), the upper size limit of “small EOs” also differed in individual articles (in the range of 59 to 100 µm in diameter). This inconsistent definition resulted in the inclusion of small to larger (vascularized) islets in different proportions, affecting the perceived contribution of small EOs to total endocrine area. By defining islets based on the presence of direct and internal vascularization, the proposed size threshold provides a more robust distinction between islets and niSSCs and is likely to clarify the contributions of these smaller endocrine structures, which have been subject to inconsistent interpretation.

## Technical aspects of islet detection

### Size of a single beta-cell

We have measured the diameter of dissociated GFP-expressing beta-cells in suspension as 15 µm (24). Within tissues either in 2D or 3D, beta-cells may appear as ~10 µm in diameter, which is a value adopted in many studies (2, 34–36). It is noteworthy that Meier et al., measured beta-cell diameter in 2D (46 subjects aged 2 weeks to 21 years) and reported a value of ~11 µm (37).

### Data analysis: 2D (area) vs 3D (volume)

2D imaging using thinly-cut sections has many useful applications, and it will continue to be valuable. However, one limitation may be the difficulty in producing accurate quantification of network structures such as vasculature (13). In 2D, blood vessels naturally appear as dots and short lines as shown in **Figure 2A**, where panels a–d are single optical slices out of 25 total. We have done an “internal/in house comparison” of 2D vs 3D quantification of capillary density (13). Each analysis was performed on approximately the same sample size (~110 islets) with a minor difference between the samples due to 2D rendering producing multiple cross-sections of a single three-dimensional islet. We then evaluated the change in the overall mean “area” ICD and islet effective diameter, followed by the average proportional change in these values among each individual islet. When using 2D renderings of the same islets, overall mean islet effective diameter and mean ICD were ~9% lower than the corresponding mean values in 3D. Evaluation of proportional differences between each 2D islet and its 3D counterpart reveals a striking contrast. Expressed as an absolute value, the discrepancy between area ICD and vICD in either direction (i.e., over-estimation or under-estimation) was 18.5%, indicating markedly decreased accuracy of measurement at the individual optical panel level. Islet effective diameter decreased by 25.2%, indicating a consistent underestimation of islet size. As shown in **Figure 2**, 2D analysis captures vICD heterogeneity and its decline with increasing islet size, but it introduces considerable proportional error at the individual islet level, potentially obscuring finer details. Mean islet diameters are systematically underestimated in 2D, because sectioning often misses the largest central plane, producing smaller, but never larger, measurements than 3D. This occurs across all islet sizes. Deviations between 2D area ICD and vICD are likely random, as vasculature appears as short lines or dots, introducing stochastic variability in vessel quantification within each section.

**Figure 2:**
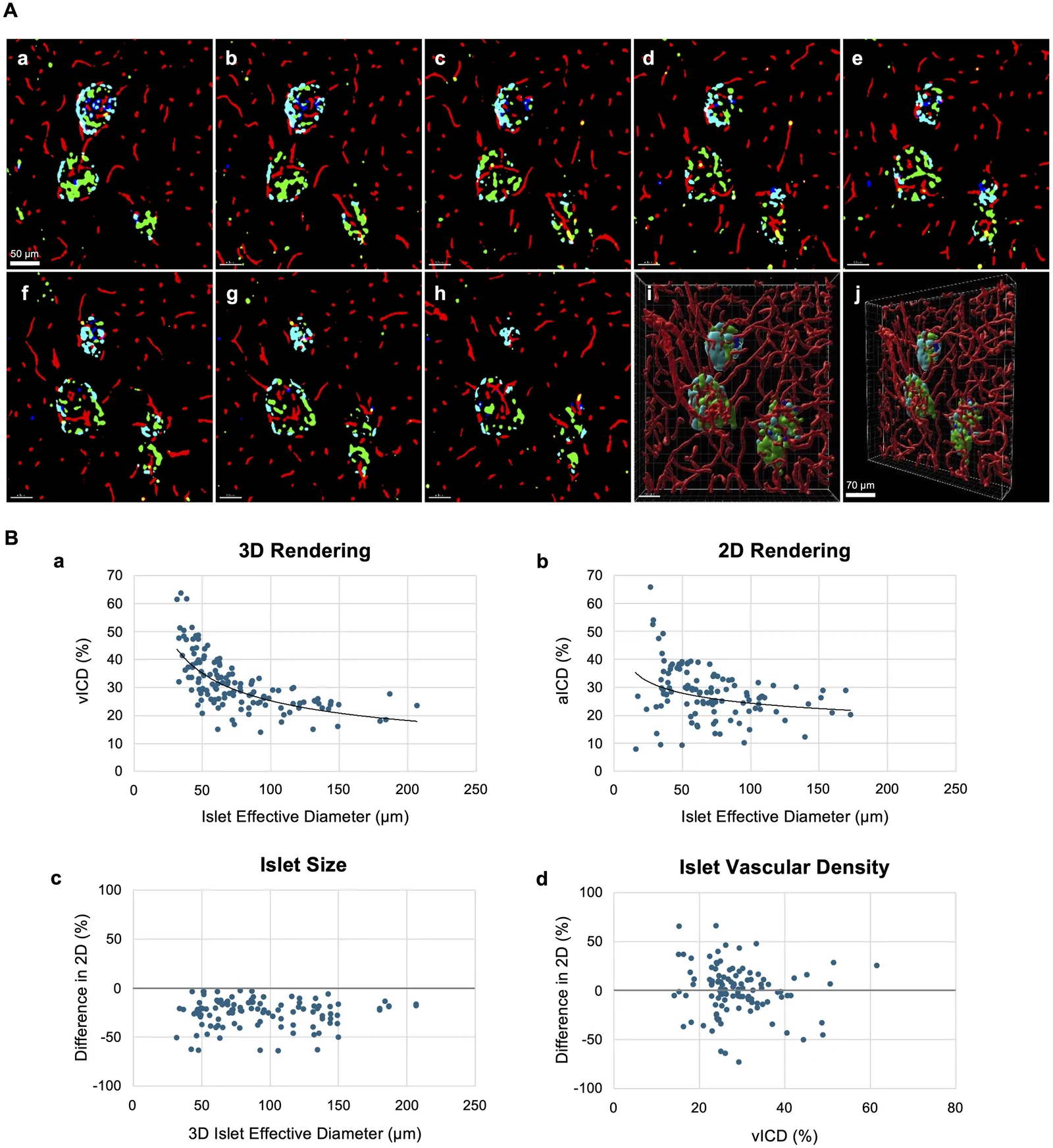
Approaches to visualizing and analyzing microvasculature microvascular analysis. **(A)** 2D vs 3D analysis. **a–h**. A series of 2D optical panels of human pancreatic tissue: insulin (green), glucagon (cyan), somatostatin (blue) and CD31 (red). A part of a stack of sequential images (out of total 25 total optical panels with 8 µm increments) is shown. **i–j**. 3D reconstruction of a stack of 2D images. Scale bar: all 50 mm except the last panel, which is 70 mm. (13) **(B)** Comparison of data acquired from the same islet sample (~120 islets) using 3D and single-slice (6 mm) to simulate 2D. **a–b**. 3D more clearly reveals the relationship between islet size and islet vascularization. **c**. Comparison of islet effective diameter between simulated 2D islets and their 3D counterparts, indicating 2D consistently underestimates islet effective diameter. **d**. Comparison of vICD in simulated 2D islets to 3D indicates inaccuracy in measurement of intraislet vascular density when omitting the full vascular network (13).

### Data presentation: diameter vs area/volume

We have been practicing the normalization of area (2D imaging) or volume (3D imaging) to effective diameter (i.e., normalized to a circle or a sphere, respectively) because diameter provides a manageable number and is a simple quantification to conceptualize (23, 33, 34, 38–41). For example, 3D size metrics such as volume or surface area should still serve well, but few would be familiar with a volume of 1.77x10–^3^ mm^3^ (or an area of 1.77x10–^2^ mm^2^), even though this is the most commonly used islet unit for islet transplantation, known as the islet equivalent (IEQ) (i.e., 150 µm in effective diameter). As indicated in our recent study of islet vascularization, the smallest and largest islets differ volumetrically by 3 to 4 orders of magnitude (13). Crucially emphasized, vICD is highly heterogenous between islets of different size, a finding that remains apparent when focusing on islet volume. However, vICD also demonstrates significant heterogeneity between islets of the same size, especially among smaller islets, <80 µm for example, where vICD ranges from 15% to 70%. When representing islet size using volume, this facet of heterogeneity is underappreciated due to the wider range of islet volume values. Furthermore, it becomes less clear that this vICD heterogeneity progressively decreases, with vICD values becoming more precise among larger islets. Islet diameter, by maintaining only a single magnitude of difference between minimum and maximum values of islet size, allows clearer view of such relationships. Therefore, diameter is advantageous for its more finite range as compared to 3D metrics. Nonetheless, it may be useful to provide a diameter-volume conversion scale.

### Statistics: are the more specimens, always the better?

Studies with a large number of samples, parameters or data points are often considered superior to smaller scale studies. How true is this principle in practice? It has been recognized that an overpowered study with an excessively large sample size can detect trivial differences as statistically significant (42–45). In clinical research, it is particularly important to incorporate measures such as the minimum clinically important difference (MCID), which distinguishes between statistically significant results and those that provide a meaningful benefit to patients (46). A recent article by Ahmed and Butt describes the two most common misconceptions about p-values: [1] the belief that they represent the probability that the null hypothesis is correct, with many researchers mistakenly believing that a p-value of 0.05 suggests a 5% chance that the outcome is a result of chance; and [2] the belief that p-values alone determine the validity or importance of a finding without accounting for effect size (47). The first misconception often leads to overconfidence in the results classified as significant, and the second may lead to the incorrect assumption that a statistically significant result automatically has clinical importance, which is not always true.

In our previous study, we observed a difference between two groups with a high statistical significance (p<0.001), yet the difference appeared biologically negligible (13). Common sense is unfortunately not a scientific term or function. Using a pooled-islet approach, our islet sample size exceeded 1,500 islets from pancreatic tissue of 16 subjects with no diabetes (ND) and type 2 diabetes (T2D). Such large samples of islets, considered best practice, proved highly useful to discern important relationships graphically, such as the relationship between volumetric intraislet capillary density (vICD) and islet effective diameter. However, our high sample size also resulted in statistically significant p-values, which to the human eye, did not appear such within the plots themselves. This ambivalence prompted our use of effect size testing, particularly Cohen’s d, to evaluate whether this difference was meaningful when adjusting for sample size. Indeed, the effect sizes were small, at 0.11 and 0.23. By contrast, significant differences in vICD between animal species and humans were found to be practically significant per effect sizes of 0.60**–**1.14, notably larger values reflecting a difference that was present in the corresponding plots. In other fields, hierarchical modeling approaches (e.g., treating islets as nested within donors) are widely used because they facilitate decomposition of observed variation into components attributable to interindividual heterogeneity and preparatory conditions. Sole reliance on a p-value derived from pooled data, in which islets are treated as independent samples, may produce misleading conclusions and foster unwarranted confidence in the findings. Studies that continue to pool data to preserve the contribution of individual islets to statistical analyses would benefit from reporting effect sizes alongside significance testing, helping to account for the influence of large islet sample sizes on statistical outcomes. Alternatively, hierarchical models/tests may obviate the need for a dual approach, particularly in studies focused on comparing groups affected and unaffected by a disease such as diabetes, rather than elucidating foundational features of islet biology.

Some datasets may be prone to overestimating differences based on biological considerations. For example, in terms of histological analyses of beta-cell/islet cell mass in the human pancreas, it has been demonstrated that there are regional differences between the head, body and tail regions (32, 39–41). With considerable individual heterogeneity, beta-cell/islet cell mass is generally highest in the tail region. The head region is anatomically unique that contains the uncinate process in which pancreatic polypeptide (PP) cells dominate the endocrine cell mass with less beta- and alpha-cells compared to other regions. Moreover, the development of the uncinate process differs in individuals (39, 48–51). Therefore, it is important to identify which region of the pancreas a given tissue section is derived from. If it is from the head region, further validation whether it is the uncinate process by PP staining is necessary. In terms of statistical evaluation, it is pivotal to be particularly cautious when using tissue sections with unknown regional origins together with “overpowered sample size,” such as a few hundred of specimens. It is possible that the intrinsic regional differences in islet cell distribution could interfere with statistical outcomes. In such studies, many specimens would not overcome the limitation but could rather amplify the underlying differences and further confound evaluation.

## Discussion

The establishment of standardized methods for quantification is essential for useful comparison of data generated by different research groups, directly facilitating the advancement of research. Furthermore, it is beneficial to explicitly characterize the differences in expected outcomes between methodological approaches to guide interpretation and ensure consistency in reporting. For example, we experimentally examined the differences of measuring beta-cell mass in 2D comparing between three approaches: [1] a large-scale analysis of the entire tissue section; [2] selecting certain numbers of islets within the section; and [3] using the point-count morphometry (41). The results indicated that both alternative methods overestimated values relative to whole-section analysis, with islet-rich area selection increasing results 3–10-fold and point-counting morphometry 4–14-fold. Importantly, this verification does not deny any methods. By recognizing systematic biases in each approach, such as a predisposition for over- or underestimation, interpretations can be made both accurate and nuanced. However, accurate quantification of network structures such as vasculature necessitates 3D analysis, since spatial distribution varies markedly between single optical sections. Islet size is consistently underestimated in 2D compared to 3D and therefore can be adjusted for, provided the analysis encompasses the entire section rather than a selective sampling of 20–30 islets. However, quantification of ICD in 2D, where capillaries appear as short line segments or dots, leads to either over- or underestimation randomly. As a result, there is unfortunately no reliable or practical method to adjust the discrepancy between 2D and 3D measurements of intraislet capillary density.

Inappropriate use of statistical power has been recognized by clinical trials that face a “yes or no” decision whether to use certain drugs or treatments for the meaningful benefit for patients. When p-values are widely used, over-reliance on significance testing could lead to misunderstanding of the results when not incorporating MCID. This is true to the preclinical basic sciences as well, particularly when dealing with large sample sizes. When p-values indicate statistical significance in highly powered studies, it is critical to use as a measure of practical significance of the result (52). Lastly, caution should be taken to recognize the marked differences in regional beta-cell/islet distributions, as well as the existence of the PP-cell rich uncinate process in the human pancreas, as this will maintain experimental accuracy in comparative studies.

New technologies keep emerging. They do not simply change tools but have a profound effect on how we observe and interpret results. Many past scientific discoveries have been re-discovered this way. Extra-islet beta-cells have repeatedly emerged as a crucial population contributing for beta-cell mass and function, which has led the field to question distinction between this population and their larger counterparts, the islets. This has even led to a challenge against “islets” as a term, introduced by Laguesse in 1893 (53) in honor of Paul Langerhans, who first described these structures in his 1869 doctoral thesis (31). Ever since, the term has been firmly linked to the field. If the issue lies in the lack of a clear size threshold for the islet (3), we propose adopting internal vascularization as their definitive criterion in contrast to capillary association for non-islets (niSSC). This approach preserves the historical and widely recognized nomenclature while providing a biologically meaningful and reproducible framework for classification.

## Scope statement

In recent publications, the term endocrine objects (EOs) have been introduced and defined as “contiguous structures containing one or more endocrine cells.” Building on this concept, a group of investigators with a strong interest in EOs convened at the IDS meeting to participate in a dedicated workshop, with the goal of developing and publishing a white paper on the topic. When the term “EO” is proposed as a potential replacement for the historically respected term “the Islet of Langerhans,” we believe that it warrants thorough and broad discussion across the field. This Perspective outlines the rationale for defining the smallest vascularized islets, thereby providing a framework for preserving the historically established and widely accepted term, the islet of Langerhans. We further discuss key considerations for standardizing islet research, including technical aspects of islet identification in two- and three-dimensional imaging, best practices for graphical data presentation, and appropriate statistical approaches for quantitative analysis. This topic is therefore timely and relevant to the need of the field.

## Data Availability

The original contributions presented in the study are included in the article/supplementary material. Further inquiries can be directed to the corresponding author.
